# Infodemiology and Infoveillance of Multiple Sclerosis in Italy

**DOI:** 10.1155/2013/924029

**Published:** 2013-08-20

**Authors:** Nicola Luigi Bragazzi

**Affiliations:** School of Public Health, Department of Health Sciences (DISSAL), University of Genoa, Via Antonio Pastore 1, 16132 Genoa, Italy

## Abstract

Multiple Sclerosis (MS) is a chronic debilitating disease of probable autoimmune inflammatory nature, whose aetiology is not fully understood, despite the many efforts and investigations. In this manuscript, we review the concept of “Multiple Sclerosis 2.0”, that is to say the Internet usage by MS patients, for seeking health and disease-related material for self-care and self-management purposes, and we introduce a Google Trends-based approach for monitoring MS-related Google queries and searches, called MS infodemiology and infoveillance. Google Trends has already proven to be reliable for infectious diseases monitoring, and here we extend its application and potentiality in the field of neurological disorders.

## 1. Introduction

### 1.1. Multiple Sclerosis 2.0

Multiple Sclerosis (MS) is a chronic debilitating disease of probable autoimmune inflammatory nature, whose aetiology is not fully understood, despite the many efforts and investigations carried out [1, 2]. Being a chronic disorder, MS has a tremendous psycho-social burden [3, 4], and recently the concept of a Web-based aid for MS patients has emerged, collecting MS-related information and at the same time trying to reduce the stressors, enhancing the self-management of the disease, facilitating the interactions between the patients and the medical team, and accurately reporting to the physician the patients' symptoms after their online registration [5].

Gunther Eysenbach coined the terms “infodemiology” and “infoveillance”, describing a new emerging approach for public health [6, 7], based on large-scale monitoring and data mining, within the conceptual framework of e-health and health Web 2.0 [8, 9]. Even if with some limitations and concerns, the Internet and the medical informatics are paving the way for new directions in the field of the epidemiological research, indicating new trends and strategies [10]. In the shift from a paternalistic medicine (P0 model) to a patient-centered approach (P6 model, where the six Ps stay personalized, preventive, predictive, participatory, psycho cognitive, and public) [11–13], patients tend to use Internet as a source of relevant health-related information, even if not all properly validated or reliable, for health education, for finding suggestions, for coping strategies, and for self-managing their disease [14]. The Internet has blurred geographical boundaries and other barriers, making it available to lay people a wealth of medical material which was rather difficult to reach before that [15]. This material could help the patients in the process of decision making, providing understandable, clear information, and decreasing their anxiety level [15]. Patients are attracted by the possibility not only to access medical material, but also to upload and create their own contents (generally known as User-Generated Content (UGC), User-Driven Content (UDC), in specific cases termed as Patient-Generated Content (PGC)), and this is opening new unprecedented avenues and scenarios, which were before unforeseeable [14, 16]. New forms and ways of communication have modified the patient-physician relationship and have introduced the so-called “Patient 2.0 phenomenon” [17].

In the “Multiple Sclerosis 2.0” era, many MS patients would accept or already regularly make use of innovative Web technologies and electronic forms of communication, as reported by Haase and collaborators [18]. The number of MS patients who use YouTube to share their experiences, to seek for advice, and to evaluate and comment on other users videos is increasing. Fernandez-Luque and collaborators [19] found that MS patients using YouTube were surprisingly informed about the latest drugs against MS. Patients share their health-related status using platforms like “PatientsLike” and other forum/blogs, and this behavior has proven to result in better clinical outcomes [20, 21]. Atreja and coworkers [22] found that MS patients judged valuable and of vital importance for them to access the Internet for MS-related information. These findings confirmed the results obtained by Hay and collaborators [23], who reported that most MS patients consulted the Web before and after the medical visit, in order to better understand the technical terms used by the physicians. They should be aware of this aspect and try to use a more patient-friendly vocabulary, and they should know which material is available on the Web, in order to react to it by providing further information or correcting some mistakes, and to discuss it with the patient. On the other hand, patients themselves are not likely to report the Internet materials to the doctors, and some of them fear that doing so would mean a lack of trust in the physicians' skill and professional competence or a challenge to their authorities [24]. Other patients experience a lack of time or time burden and constraints during the visits [15]. Most patients, indeed, do not frequently bring the information surfed on the Internet to their clinic visits, that is to say sharing that material with their doctors or asking them questions based on the Web searches [15]. Moreover, doctors rarely advice their patients to search health-related material on the Internet [15]. Lejbkowicz and collaborators [25] found that most MS patients consult online information and that the Internet usage positively correlates with the MS status, severity, and degree of disability. Moreover, they consider online information as reliable but more accessible and understandable than the book and manual materials, and thus beneficial, without harmful effects. Marrie and coworkers [26] found similar results, and using a logistic regression model and younger age, less degree of disability, higher annual earning were predictor of the Internet usage. Therefore, neurologists should interact with the patients and provide them with a detached report of the effective quality of the Web-based material and refer them to specific websites, as the Information Rx project (http://www.medlineplus.com/)—a joint venture from the American College of Physicians and the National Library of Medicine (NLM)—recommends to explore different and new information channels. To better discuss with MS patients, doctors should know which are the most frequent hit searches and their needs or knowledge gaps [27]. For this purpose, Google Trends could play a major role.

### 1.2. Google Trends

Google Trends is a Web application that enables to visualize hit searches volumes [28]. Indeed, the Google-based approach has recently emerged as a new tool in the field of infodemiology and infoveillance [29]. It is particularly useful to monitor especially influenza epidemics [30–33]. SARS was discovered through monitoring search engines, since Larry Brilliant discovered a “viral” search of anti-influenza drugs in China [34].

Scholars have stressed the correlations between Internet search and the triage data, the hospital access, and the need of drugs [35, 36].

Google Trends has been usually used for monitoring infectious diseases (from influenza to tuberculosis and other emerging or drug-resistant infectious strains) [37, 38], but its enormous potentiality and applications in other fields of medicine have been noticed only recently. Gunn III and Lester surveyed suicides using search engines [39], and other scholars have replicated these findings [40, 41], while Deluca and collaborators exploited the Web for studying the epidemiology of drug addiction [42]. Internet key-word searching has been used also in the field of human sexuality, trying to better understand the dating and mating behaviors [43, 44]. another interesting application of Web hit search is the real-time detection of kidney stone diseases [45], but so far, to our knowledge, no one has explored the possibility of monitoring Google searches that refer to MS and associated concepts.

## 2. Materials and Methods

### 2.1. Google Trends

Google Trends, an online tracking system of Internet hit search volumes, recently merged with its sister project Google Insights for Search, which was accessed from 2004 to 2012, since data before 2004 were not available on searching for “sclerosi multipla” (Italian for MS) [28]. All the queries have been downloaded and analyzed. It is noteworthy to remember that Google Trends provides the user data that are scaled and normalized in such a way that the numbers reflect how many searches have been performed for a particular term or category, relative to the total number of queries carried out on Google over time, rather than representing absolute search volume numbers. For this reason, data are analyzed as NFV (normalized flux volume) or NSV (normalized search volume). Moreover, in order to avoid any bias in treating and manipulating the data, these have been rescaled and renormalized, taking into account the Digital Divide (North-South gradient in the access to the Internet and different Internet usages over the time). Corrections have been applied, using data from the ISTAT (Italian National Institute of Statistics). This has ensured robust findings.

### 2.2. Statistical Analysis

Autocorrelation is the correlation of a parameter with itself over the time; autocorrelation functions of this series of data, both crude and adjusted partial, were computed using R environment [46]. The wavelet transform of the time series and the wavelet power spectrum (WPS) analysis were carried out using algorithms written in Matlab accessible on http://paos.colorado.edu/research/wavelets/ [47, 48]. The multiple linear regression fitting was performed in R environment [49]. A list of MS symptoms and MS-related terms was searched in Italian language and their flux volumes were correlated with the MS hit search data. Pearson's correlation coefficient, that is to say the measure of the linear correlation between two variables, was calculated with SPSS software V21.0.0 package (IBM) and using R environment [50]. *P*-values were computed with SPSS, and values equal or less than 0.05 were considered statistically significant.

## 3. Results

### 3.1. MS Time Series

**Figure 1:**
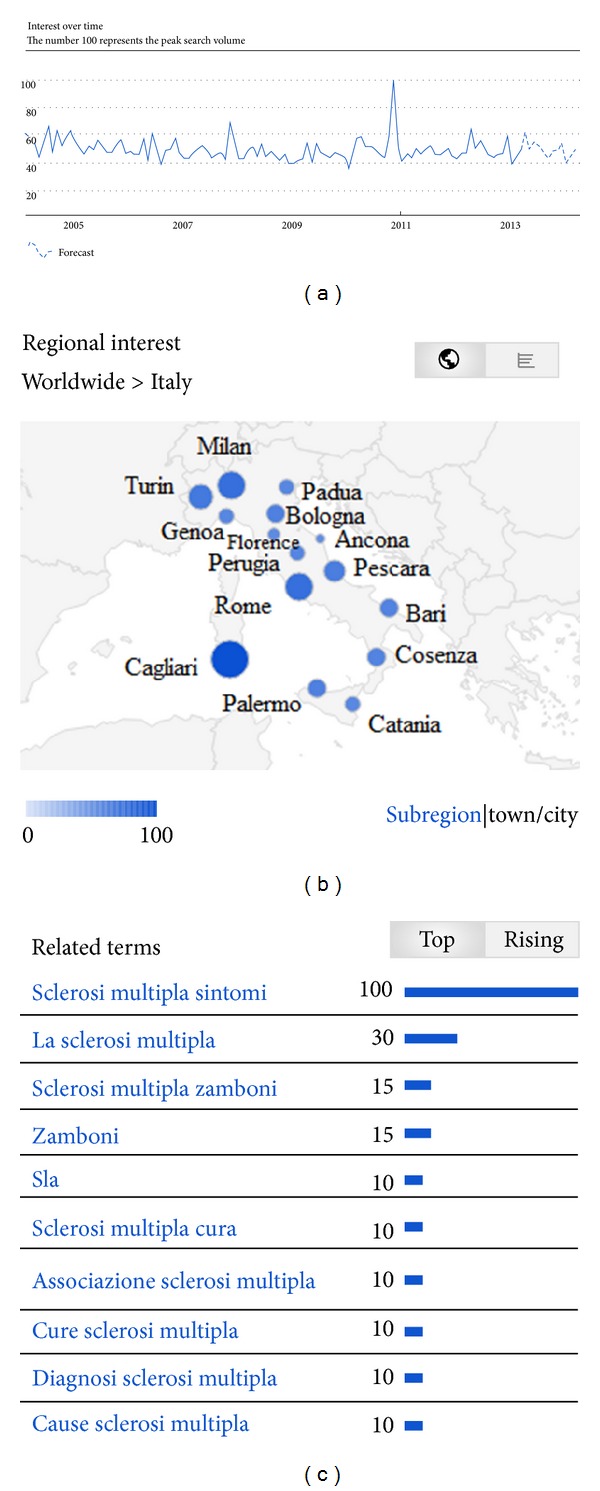
Google Trends-based MS hit search volume over the years from 2004 to 2012 and forecast trend projected over 2013 (a), a map of labeled Italian towns with higher flux volumes (b), and a list of the most searched MS-related terms (c).

**Figure 2:**
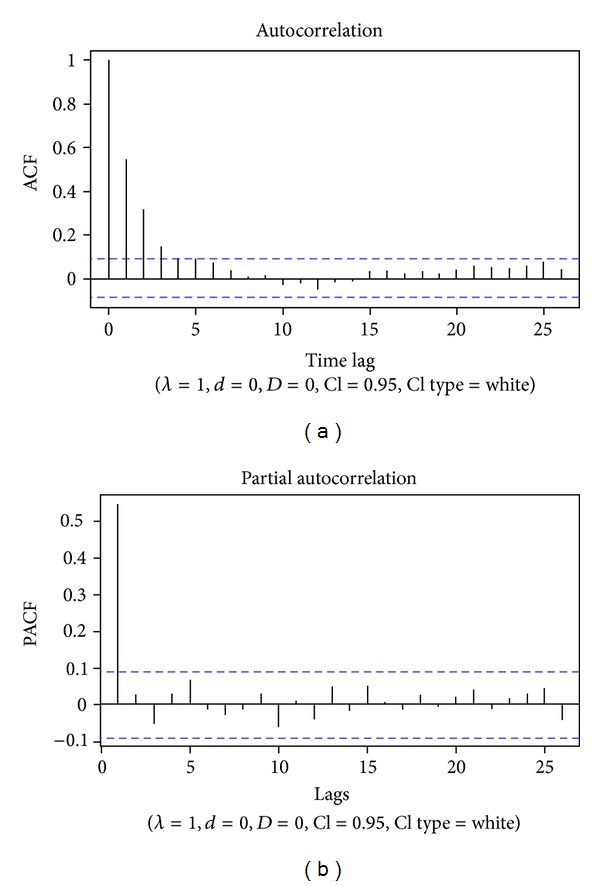
Autocorrelation plot for the MS hit search (a) and partial autocorrelation plot (b), showing no annual cyclical pattern or regular trend.

**Figure 3:**
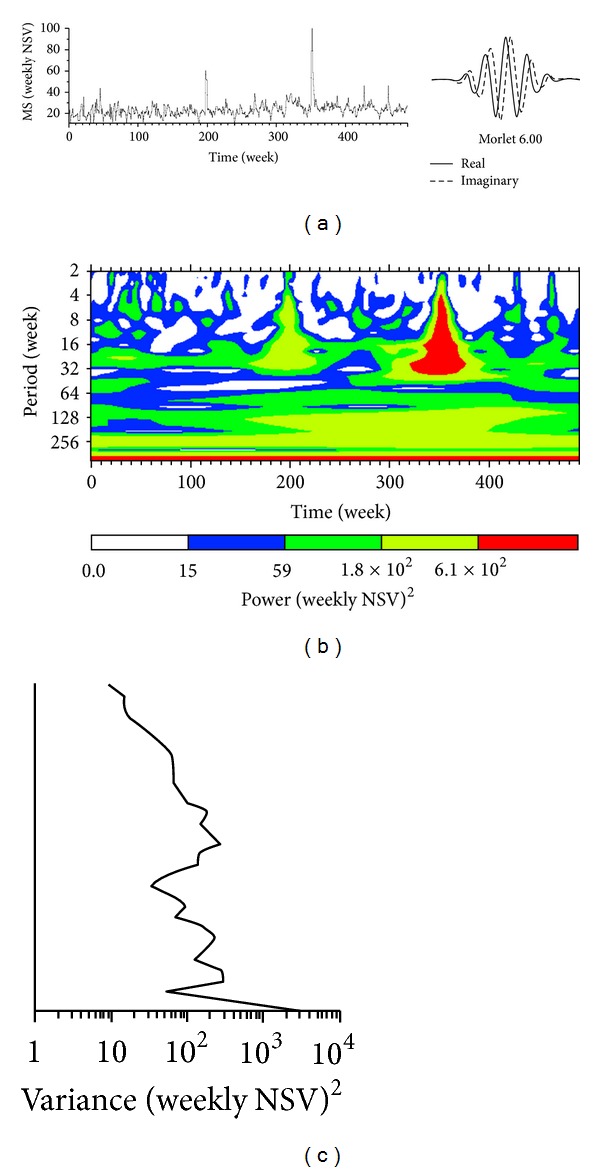
Exploratory spectral analysis, showing (a) MS time series and (b) the wavelet power spectrum (WPS) analysis. The contour levels are chosen so that 75%, 50%, 25%, and 5% of the wavelet power are above each level, respectively. (c) The global wavelet power spectrum (GWPS) analysis. These analysis were carried out using algorithms written in Matlab and accessible on http://paos.colorado.edu/research/wavelets/. Time unit is week; NSV stays for normalized search volume.

**Figure 4:**
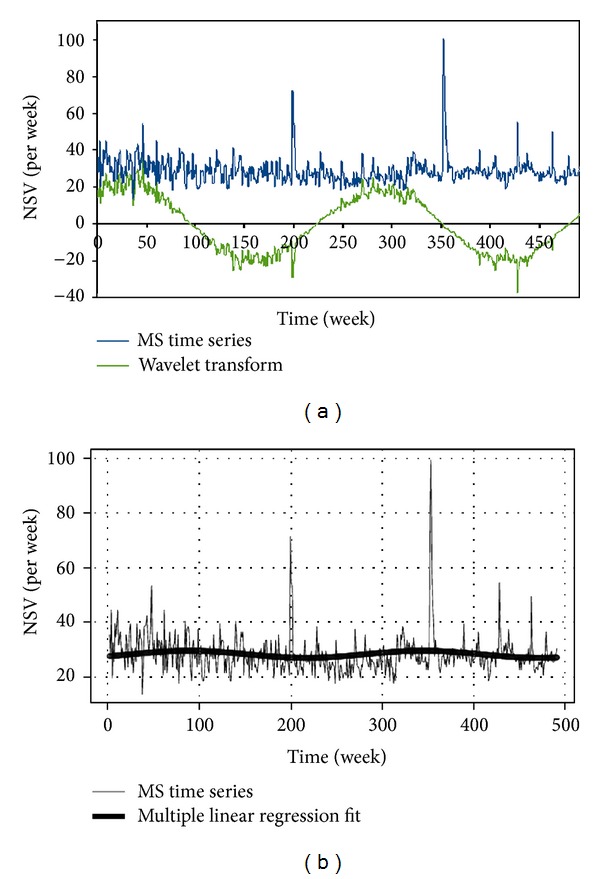
(a) The wavelet transform of the MS time series data. (b) The multiple linear regression fit of the MS time series data.

The pattern of Internet search volume (Figure 1) did not reveal a cyclic trend, as can be seen from the autocorrelation diagram (Figure 2). No annual or seasonal trends were found. The flux volume remained constant from 2004 to 2012, apart from a peak in 2007-2008 and another one in 2011-2012. However, multiannual (4-5 years) long-term trends were revealed by the exploratory analysis carried out with the WPS technique (Figure 3) and confirmed by the multiple linear regression (*P* value 0.023, intercept 28.76, sine-regression coefficient 1.07, and cosine-regression coefficient −0.73) (Figures 4(a) and 4(b)). 

### 3.2. MS-Related Google Trends Queries

**Figure 5:**
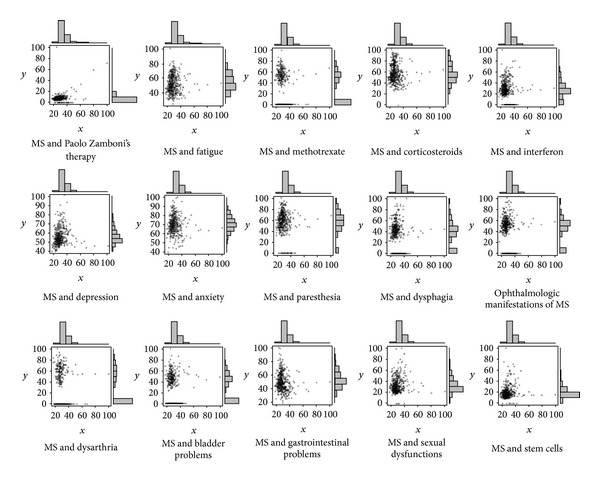
Pearson's correlation plots for some of the most important MS-related hit search terms.

MS therapy and symptoms are the most searched MS-related terms by the users. In most cases, the Pearson's correlation coefficients yielded a statistical significance (*P*-value <0.05) (Figure 5, Table 2). Some of these correlations have a positive sign, like MS and sexual dysfunctions, MS and depression (for the category of MS and symptoms), MS and Zamboni's intervention, MS and stem cells (for the category of MS and treatments/therapy), while others a negative one (viz., MS and interferon for the category of MS and treatments/therapy; MS and paresthesia; MS and dysphagia; MS and ophthalmologic symptoms; MS and gastrointestinal symptoms for the category of MS and symptoms).

## 4. Discussion

### 4.1. MS Time Series

The main finding of this article is the feasibility of adopting a Google Trends-based survey for MS. The results have been compared with those present in the literature (about epidemiology, both temporal and spatial, and the Internet usage) and have been validated. 

Two main peaks can be observed in the time series. The first one is related to the public disclosure of Nicoletta Mantovani of being suffering from MS. The second observed peak may be correlated with the introduction of Paolo Zamboni's therapy, which attracted a lot of media and layed public interest. Paolo Zamboni's therapy is a controversial surgical operation, devoted to cure the Chronic Cerebrospinal Venous Insufficiency (CCSVI), a new nosological entity which Zamboni suggested to be the cause or one of the causes of MS in a high percentage of MS patients [51]. As noticed by Vera et al. [52] and by Machan and collaborators [53], the number of online resources and material related to CCSVI is increasing, and peer groups are discussing this opportunity on social networks and dedicated forums. Nicoletta Mantovani herself announced she was healed after undergoing Zamboni's operation. Apart from these peaks, that can be considered as transitory bursts, the search volume remained quite constant. Other studies have validated this finding, confirming that media interests do not perturb time series which are rather robust (see, e.g., [54] and references therein). 

It is controversial whether the prevalence and the incidence of MS have remained unvaried or increased in the last years, since some studies report an observed increase [55–58], while other fail to replicate these findings [59]. Some scholars have argued that the increase could be explained taking into account environmental factors, while others have stated that it is only an apparent increase which may be due to confounding biases, better diagnostic criteria and therefore better case ascertainment, and longer survival rates as well as newly introduced diagnostic criteria and definitions, incorporating more sophisticated and particularly sensitive para-clinical and preclinical tools, and availability and accessibility of qualified structures and personnel [60]. However, Google Trends data were not available before 2004 and we failed to investigate MS-related queries over a longer range.

It is interesting to notice that the Italian towns with the higher searching fluxes and volumes include Cagliari, and Sardinia is a well-known high risk area [61, 62] (Figure 1, Table 1). The other towns are equally spatially distributed; that is to say we do not observe any geographical gradient between North and South in terms of Google Trends query and search fluxes and volumes, as confirmed by many detailed epidemiological Italian surveys, which have proven to be against the “latitude theory” introduced by Millefiorini et al. [63]. However, it must be noticed that some scholars while criticizing the latitudinal gradient theory for incidence maintain that the latitudinal gradient theory for prevalence may be still valid [64].

### 4.2. MS Google Trends Queries

We divided the most search terms in two categories, namely, and MS and symptoms, MS and treatments/therapy. This finding confirms a research carried out by Lejbkowicz and collaborators [25] that “understanding the disease” and “treatments” were the most viewed topics. Even though statistically significant, each MS-related term has a low Pearson's correlation coefficient, and for this reason only a panel of several words reach a good overall coefficient. This suggests that MS patients would not be limited to search for a few words, but for an array of terms. The different signs (minus and plus) of the correlations may reflect a different time pattern of MS-related queries and could help the clinicians in better understanding the patients' needs and requests.

### 4.3. Limitations

However, this study has some limitations. For example, we monitored the trend of the Internet searches volumes over the time, but we did not investigate the completeness nor the reliability of the Web material related to MS. For these reasons, this study should be complemented with a content analysis of the Web sites and with *ad hoc* surveys to MS patients, asking them their understanding and their reaction to the online material and information, as well as their Internet usage. Actually, this is a serious gap in the current Italian research. It would be clinically important to design specific Web content, in order to meet the patients needs, requests, and expectations. Moreover, this could pave the way for interventions made directly by the practitioners to interact with the patients, to post high-quality material dedicated to MS symptoms and treatments, and answer patients' doubts and questions. Lowe-Strong and McCullagh [65] implemented a Web Portal endowed with a user-friendly visual interface for pain and MS-related symptoms self-recording, while other sites enabling the self-monitoring of MS symptoms, prescriptions orders, laboratory and clinical results retrieval, online patient education, news and updates on MS research, and a timely communication with the medical staff are being designed [66]. Recently, Jones and coworkers have created a dynamic interactive Web Portal, where MS patients can record their symptoms and the quality of life [67]. Boeschoten and collaborators [68] developed a Web-based problem-solving treatment (PST) specifically designed for MS patients suffering from depressive symptoms.

## 5. Conclusion

In this contribution, we showed that a Google Trends-based infodemiology and infoveillance system could be successfully applied also to monitor MS-related information and Google queries and probably to other chronic degenerative disorders, and not only to infectious diseases. Even with its warranted limitations, Web-based epidemiology has a promising potentiality.

## Figures and Tables

**Table 1:** A list of the towns (first column) with the higher normalized MS hit search flux volumes (NFV), as provided by Google Trends. In the third column, these crude NFV data have been rescaled taking into account the Digital Divide and North-South access to the Internet (according to the ISTAT) and re-normalized. We term these data the adjusted NFV.

City/town	Crude NFV (%)	Adjusted NFV (%)
Cagliari	100	100
Rome	80	93
Milan	80	93
Turin	74	86
Pescara	71	82
Palermo	69	66
Bologna	69	80
Bari	67	64
Cosenza	67	64
Perugia	66	77
Padua	65	75
Genoa	65	75
Catania	64	61
Florence	62	72
Ancona	60	70

**Table 2:** Pearson's correlation coefficients for different hit search volumes related to MS and their statistical significance according to their two tails *P* value.

Hit search volume	Pearson's correlation coefficient	*P* value (two-tails)
MS and symptoms		
MS and fatigue	*r* = 0.02	*P* = 0.66
MS and depression	*r* = 0.11	*P* = 0.02*
MS and anxiety	*r* = −0.04	*P* = 0.44
MS and paresthesia	*r* = −0.11	*P* = 0.01*
MS and dysphagia	*r* = −0.10	*P* = 0.03*
Ophthalmologic manifestations of MS	*r* = −0.12	*P* = 0.01*
MS and dysarthria	*r* = −0.00	*P* = 0.96
MS and bladder problems	*r* = 0.04	*P* = 0.36
MS and gastrointestinal problems	*r* = −0.14	*P* = 0.00*
MS and sexual dysfunction	*r* = 0.10	*P* = 0.03*

MS and treatments/therapy		
MS and Paolo Zamboni's therapy	*r* = 0.35	*P* = 0.00*
MS and stem cells	*r* = 0.12	*P* = 0.01*
MS and methotrexate	*r* = −0.09	*P* = 0.05
MS and corticosteroids	*r* = −0.07	*P* = 0.15
MS and interferon	*r* = −0.10	*P* = 0.04*

*Statistically significant.
